# miR-204 Targeting of *Ankrd13A* Controls Both Mesenchymal Neural Crest and Lens Cell Migration

**DOI:** 10.1371/journal.pone.0061099

**Published:** 2013-04-19

**Authors:** Raffaella Avellino, Sabrina Carrella, Marinella Pirozzi, Maurizio Risolino, Francesco Giuseppe Salierno, Paola Franco, Patrizia Stoppelli, Pasquale Verde, Sandro Banfi, Ivan Conte

**Affiliations:** 1 Telethon Institute of Genetics and Medicine, Naples, Italy; 2 Institute of Genetics and Biophysics, Naples, Italy; 3 Medical Genetics, Department of Biochemistry, Biophysics and General Pathology, Second University of Naples, Naples, Italy; University of Colorado, Boulder, United States of America

## Abstract

Loss of cell adhesion and enhancement of cell motility contribute to epithelial-to-mesenchymal transition during development. These processes are related to a) rearrangement of cell-cell and cell-substrate adhesion molecules; b) cross talk between extra-cellular matrix and internal cytoskeleton through focal adhesion molecules. Focal adhesions are stringently regulated transient structures implicated in cell adhesion, spreading and motility during tissue development. Importantly, despite the extensive elucidation of the molecular composition of focal adhesions, the complex regulation of their dynamics is largely unclear. Here, we demonstrate, using live-imaging in medaka, that the microRNA miR-204 promotes both mesenchymal neural crest and lens cell migration and elongation. Overexpression of miR-204 results in upregulated cell motility, while morpholino-mediated ablation of miR-204 activity causes abnormal lens morphogenesis and neural crest cell mislocalization. Using a variety of *in vivo* and *in vitro* approaches, we demonstrate that these actions are mediated by the direct targeting of the *Ankrd13A* gene, which in turn controls focal cell adhesion formation and distribution. Significantly, *in vivo* restoration of abnormally elevated levels of *Ankrd13A* resulting from miR-204 inactivation rescued the aberrant lens phenotype in medaka fish. These data uncover, for the first time *in vivo,* the role of a microRNA in developmental control of mesenchymal cell migration and highlight miR-204 as a “master regulator” of the molecular networks that regulate lens morphogenesis in vertebrates.

## Introduction

Cell migration plays a key roles in embryo morphogenesis [1]. Migrating cells are often classified into two groups, namely a) epithelial cells, which move in a coordinated manner and maintain their cohesive contacts during tissue patterning [2] and b) mesenchymal cells that loose cell-cell contacts and undergo solitary migration, controlled by the cross talk between the extra-cellular matrix and the internal cytoskeleton through focal adhesion (FA) molecules [3]. Coordinated and dynamic regulation of FA complexes and the actin structures with which they are associated are critical for cell proliferation, survival, cell adhesion and motility [4]. Specific macromolecular structures in FAs are reiteratively assembled and disassembled in cells, principally due to multiple binding sites that are contained in most components of FAs, to control the subsequent cellular motility and adhesion programs [5], [6], [7]. Most of these cellular processes are critically sensitive to FA composition, and variations in the normal level of each FA proteins appear to result in a variety of cellular motility, spreading and adhesion anomalies [8]. However, the mechanisms underlying the precise expression regulation of each FA components are still poorly understood and probably involve additional molecular elements.

MicroRNAs (miRNAs), a class of 20- to 25-nucleotide small noncoding RNA molecules with a basic role in post-transcriptional regulation of gene expression [9], are expected to play critical roles in the control of cell migration and adhesion and to directly contribute to extracellular matrix (ECM) remodeling [10]. However, in spite of a number of reports that document the role of miRNAs in the regulation of cell migration and the posttranscriptional control of cell adhesion molecules *in vitro* [10], [11], [12], the mechanisms of miRNA-dependent control of cell migration in living organisms remain currently elusive.

MiR-204 displays an evolutionary conserved predominant expression in the eye, particularly in the retinal pigment epithelium (RPE), lens, ciliary body, retina and migratory neural crest cells (NCCs) [13], [14]. Interestingly, through *in vitro* studies, miR-204 has been associated with cell migration and invasiveness [15], [16], [17], [18], [19], [20]. However, it is completely unknown whether this miRNA has a similar function in cells of living organisms. Our previous work showed that miR-204, by modulating the expression of the *Meis2* target gene, plays a role in the control of lens and retinal development in the medaka fish model organism [13] and presumably has a similar role in other vertebrates (although this remains to be demonstrated). Notably, knockdown of miR-204 in medaka fish led to mislocalization of lens fiber cells during embryo development [13]. However, the activity of miR-204 in cell migration, and its specific influence on lens fiber cell organization is not Meis2-dependent [13] and has not been elucidated yet. Lens epithelial cells derive from the surface ectoderm and differentiate into elongated mesenchymal-like lens fiber cells by transcriptional reprogramming, as shown by the activation of type I collagen and α-smooth muscle actin expression (α-SMA) during differentiation [21], [22], [23]. This differentiation process is coupled with directed migration and modification of both cell-cell and cell-matrix adhesion properties [24]. Here, we investigated *in vivo* the function of miR-204 in cell migration. By combining a variety of gain- and loss-of-function approaches, we demonstrate that miR-204 controls the migration of mesenchymal-like lens fiber cells and NCCs. Our results indicate that, in exerting this action, miR-204 regulates FA formation through direct targeting of the *Ankrd13A* gene. This study sheds further light on the functional role of miR-204 in eye development and provides support to the contribution of miRNA activity in the regulation of mesenchymal cell migration *in vivo*.

## Materials and Methods

### Medaka Manipulation and Analysis

#### Ethics statement

All studies on fish were conducted in strict accordance with the institutional guidelines for animal research and approved by the Italian Ministry of Health; Department of Public Health, Animal Health, Nutrition and Food Safety in accordance to the law on animal experimentation (article 7; D.L. 116/92; protocol number: 00001/08/IGB; approval date Oct. 22, 2008). Furthermore, all animal treatments were reviewed and approved in advance by the Ethics Committee of the Institute of Genetics and Biophysics, IGB Animal House, (Naples, Italy).

#### Medaka fish

Both morpholino (Gene Tools, LLC) and miR-204 mimics (Dharmacon) injections, and whole-mount RNA in situ hybridization on medaka fish were performed as described previously [13]. Morpholinos (Gene Tools, LLC, OR) were designed and injected into one-cell fertilized embryos. Specificity and inhibitory efficiency of each of morpholinos were determined as described [13]. Optimal Mo (0,09 mM) concentration was determined on the basis of morphological criteria. miRIDIAN Dharmacon microRNA Mimics for miR-204 were injected at a final concentration of 4 µM. Controls for the Mo and mimic injections were performed as described [13]. Templates information are provided in Table S1. The Cab inbred medaka strain were kept and staged as described [13], cell tracking in the living *olTrpm1:*GFP transgenic medaka lines was monitored as described [25] (see also supplemental Materials).

### Gene Silencing, Quantitative Real-time PCR and Luciferase Assays

H36CE human lens epithelial (a generous gift of Dr. Paola Bovolenta, Universidad Autónoma de Madrid) [26] and A549 non-small cell lung carcinoma cell lines [27] were cultured at 37°C in a humidified chamber supplemented with 5% CO2 as previously described [26], [27]. Cell transfections and quantitative RT-polymerase chain reaction (qRT-PCR) experiments were performed as described [13]. Cells were transfected with either 50 nM concentration of miRIDIAN™ Dharmacon microRNA Mimics (miR-204 or negative control cel-miR-67) or 80 nM miRIDIAN™ Dharmacon microRNA Inhibitor (miR-204 or negative control cel-miR-67). Plasmids containing either the wild-type 3′ UTR sequence or their mutated version, containing three point mutations in the seed of the predicted miR-204 target site, and psiUx plasmid constructs containing the hsa-pre-miR-204 sequence were used in Luc assays, as described previously [13]. For gene silencing, H36CE cells were transfected with ON-TARGET plus SMARTpool siRNAs (Ankrd13a and negative control, Euroclone) at a final concentration of 150nM. For overexpression experiments, the ANKRD13A cDNA was amplified and inserted in frame into the p3×FLAG-CMV™-14 C-terminal (Sigma) expression vector at the KpnI restriction site. pAnkrd13a-3×Flag was transfected with Lipofectamine 2000 (Invitrogen) following the manufacturer’s protocol. Cells were fixed after 48 h for immunolabeling staining or harvested after 48 h for both total RNA and protein extraction. The 3′-UTR of the human ANKRD13A gene containing the miR-204 target site (Fig. S5E), was cloned downstream the coding region of the Luciferase (Luc) reporter gene, and tested the ability of miR-204 to affect Luc activity in vitro. The adhesion assays and live imaging for the wound healing assay on both transfected H36CE and A549 cell lines was performed using an inverted microscope system (Leica DMI6000) as previously described [28]. The primers used are listed in Table S1.

### DNA Content Analysis and Flow Cytometry

H36CE transfected cells were disaggregated and PI staining carried out. DNA content was analyzed on a BD FACSAria and results processed with BD software. Each assay was performed in duplicate and all results are shown as mean ± SD for at least three independent assays. A χ2 test was used for statistical data analysis.

### Western Blot and Immunolabeling

Immunoblotting and immunolabeling were performed as described [29], [30]. The following antibodies were used: anti-flag M2-peroxidase and anti-β-tubulin mouse monoclonal antibodies (1∶1000 and 1∶250; Sigma); anti-Fak mouse monoclonal antibody (1∶100; Cell Signaling); rhodamine-phalloidin (1∶1000; Sigma); anti-Flag rabbit polyclonal antibody (1∶500; Sigma). Alexa-488–conjugated goat anti-rabbit or anti-mouse (1∶1,000; Invitrogen) IgGs were used as secondary antibodies. DAPI staining was performed for nuclei. Confocal images were acquired using Zeiss (LSM 710).

### Time-lapse Imaging for Cell Tracking in Living Medaka Embryos

The analysis was carried out using a LEICA AF6000 LX multiposition advanced fluorescence imaging and live cell analysis system (Wetzlar, Germany). The live imaging was performed using an inverted microscope system (Leica DMI6000) at 27°C. Z-stacks were recorded every 5 minutes by scanning areas of 436.8×332.8 µm (0.65 µm pixel-1) with 2 µm steps over a total distance of 98 µm. The 4D captured images thus obtained were montaged using the software package AF6000 (Leica). Maximum intensity projection of Z-stacks was done for 4D images.

### Computational Analysis and Statistics

miR-204 target predictions were obtained using the TargetScan, miRanda and PicTar algorithms [31], [32], [33]. Gene Ontology (GO) analysis was performed using the DAVID tool [34]. Data are expressed as means ± standard deviations and represent one of at least three separate experiments undertaken in triplicate, unless stated otherwise. Differences between data sets were determined by the Student *t* test. Differences described as significant in the text correspond to *P* values of <0.05.

## Results

### miR-204 Controls Motility of Mesenchymal Cells

**Figure 1:**
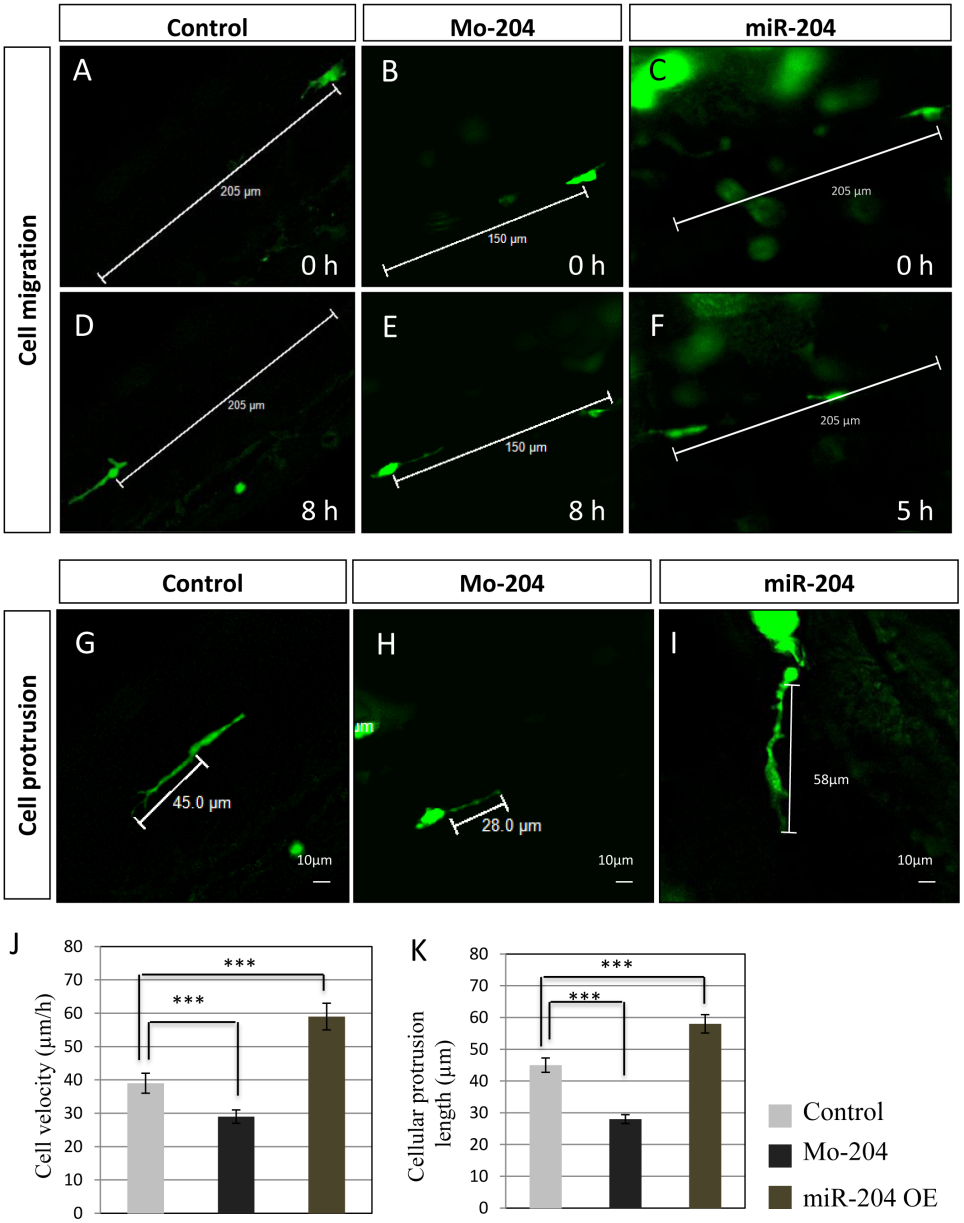
miR-204 controls mesenchymal NCC migration *in vivo*. (A–F) Expression of the *olTrpm1*:GFP transgene in migrating NCC of control, miR-204 MO, and miR-204 OE medaka embryos. Representative images from time-lapsed movie of *olTrpm1*:GFP expression in the trunk from st24 (A, B, C) to St25 (D, E, F; 8 hrs later), dorsal view (see also Movies in the supplementary material). (G–I) Time-lapse analysis showing cellular protrusion of migrating NCCs in control (G), miR-204 MO (H), and miR-204 OE (I) medaka embryos. Notably, Mo-miR-204 induces a shorter cellular protrusion (H), whereas miR-204 OE NCCs show longer protrusions (I) when compared to control embryos (G). (J) Tracking the position of the migrating NCCs revealed that the velocity of NCC migration was decreased in MO and increased in miR-204 OE embryos. (K) Analysis of maximum extension of the cellular protrusion of migrating NCCs revealed that the projection cell lengths were decreased in MO and increased in miR-204 OE embryos. Bars in J and K are means ± SEM of values; ***P<0.0001 (t tests). Images are representative of at least 5 time-lapse movies from 10 independent experiments.

Our previous studies suggested that miR-204 [NR_029621.1] affects lens and retinal development by repressing *Meis2* [NM_001159569.1] and its transcriptional target *Pax6* [NM_001244202.1] [13]. During characterization of morphological changes induced by miR-204 overexpression, we observed that in miR-204 morphant (Mo-miR-204) embryos, that lens fiber cells are misplaced and disorganized in the center of the lens vesicle, while, in control embryos, they are elongated and form organized concentrical layers (Figure S1). Furthermore, we observed an abnormal organization along the ventro-dorsal axis of the lens epithelium and lens fibers, which are normally distributed along the proximo-distal axis (Figure S1). We also found that miR-204 knockdown determined the misplacement of mesenchymal NCCs, but not of RPE cells (Figure S1 and data not shown), thus suggesting a possible role of miR-204 in posttranscriptional control of mesenchymal cell migration. To gain insight into the abnormal localization of miR-204-expressing migratory cells, we carried out a detailed analysis of the observed phenotype. First, we examined the migratory activity of miR-204-expressing NCCs by *in vivo* live imaging in medaka at single-cell resolution. The *Trpm1* gene [NM_020952.4], which harbors the miR-204 locus in its intron, was previously suggested to be co-expressed with its hosted miRNA in vertebrates [35]. Thus, we decided to specifically mark NCCs by green fluorescent protein (GFP) in an *olTrpm1*:GFP medaka transgenic line (Figure S1 and data not shown). We observed that in Mo-miR-204 embryos, NCCs showed a decreased migration speed with respect to control embryos (Figure 1A, B, D, E, J–K and movies S1 and S2). This migratory delay was accompanied by a decrease in the length of cellular protrusions (Figure 1G–H). In contrast, in embryos that overexpress miR-204 (OE), NCCs migrated faster than in controls and showed longer cellular protrusions (Figure 1A, C, D, F, G, I, J–K; and movie S3). Therefore, these findings suggest that miR-204 controls the migration and localization of mesenchymal NCCs *in vivo*. To further corroborate the latter result, we analyzed the effect of miR-204 expression perturbation on the migration of lens cells by wound-healing assays [36]. Towards this goal, we decided the use an appropriate *in vitro* system, namely the human H36CE lens cell line. We found that miR-204 knockdown (KD) in H36CE cells significantly reduced cell migration (Figure S2). On the contrary, miR-204 OE resulted in an increase of cell migration vs. control cells (Figure S2). Consistent with a specific effect on cell motility, no significant difference in cell cycle distribution was observed in either miR-204 KD nor OE cells compared to control cells (Figure S3). As control, we observed that perturbation of miR-204 activity led to the expected expression changes of Meis2 and of multiple lens differentiation markers (Figure S3), as previously observed in medaka embryos [13].

To understand if miR-204 exerts a pro-migratory activity on both epithelial and mesenchymal cells, we performed wound-healing assays in a lung tumor cell line (A549) in which full epithelial to mesenchymal transition (EMT) can be triggered by TGF-beta (TGFB1) treatment [27], [37]. However, no change in the miR-204 expression level was observed in the A549 cell line after TGF-beta treatment (data not show). Interestingly, we found that the motility of the TGFB1-treated A549 cells, which behave as mesenchymal fibroblastoid cells, was strongly induced in response to miR-204 OE, while miR-204 overexpression had no effect on the motility of untreated cells, which retain an epithelial status (Figure S4). These results suggest that miR-204 plays a role in mesenchymal-type single-cell migration during vertebrate embryo development.

### MiR-204 Controls Focal Adhesion Organization

**Figure 2:**
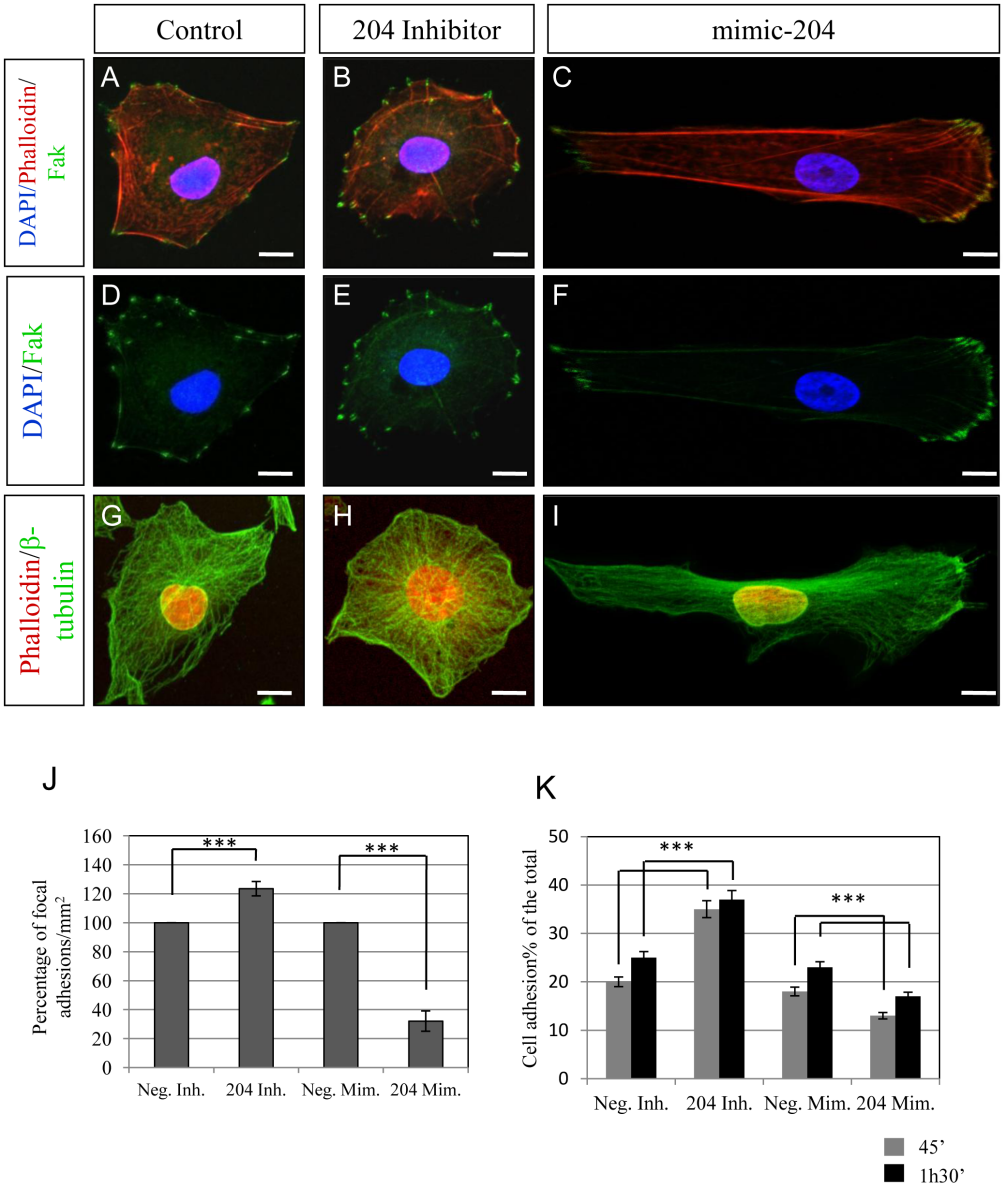
miR-204 affects cytoskeleton and FA organization. Immunostaining of actin filaments (Phalloidin, red), FA (Fak, green) and nuclei (DAPI-blue) in control (A, D), miR-204 KD (B, E), and miR-204 OE (C, F) H36CE lens cells. Immunostaining of β-tubulin (green) and nuclei (Propidium Iodide (PI), red) in control (G), miR-204 KD (H), and miR-204 OE (I) H36CE lens cells. (J) miR-204 depletion significantly increases the number of FA (indicated as percentage/mm^2^) in H36CE cells with respect to control. A marked decrease of both percentage of FA per area and FA polarized distribution was observed in miR-204 OE H36CE lens cells. ***P<0.0001 (t tests). K Extent of cell adhesion was plotted as a percentage. miR-204 OE significantly decrease the number of cell adhesion. A marked increase of this percentage was observed in miR-204 KD H36CE lens cells. ***P<0.0001 (t tests).

Polarized organization of FA and fasciculated architecture of the cytoskeleton filaments represent key mark modifications associated with cell motility [38], [39]. Therefore, we investigated how miR-204 controls lens cell motility by analyzing the organization of the actin cytoskeleton and distribution of FA in both miR-204 KD and OE cells compared to control cells. Notably, we observed that miR-204 KD in H36CE cells determined dramatic morphological alterations, i.e., a more round and compact aspect of the cells (Figure 2). These changes were accompanied by a significant disassembly of fasciculated actin, more radial distribution of microtubules and an increase of FA that resulted in enhancement of cell adhesion (Figure 2A, B, D, E, G, H, J–K). The opposite phenotype was observed in miR-204 OE cells (Figure 2A, C, D, F, G, I, J–K), which showed fasciculated microtubules and actin and a polarized distribution of FA. Therefore, we demonstrated that miR-204 promotes cell migration by determining drastic changes of cell morphology, cytoskeleton rearrangements and redistribution of FAs.

### *Ankrd13A* is a Direct Target of miR-204

**Figure 3:**
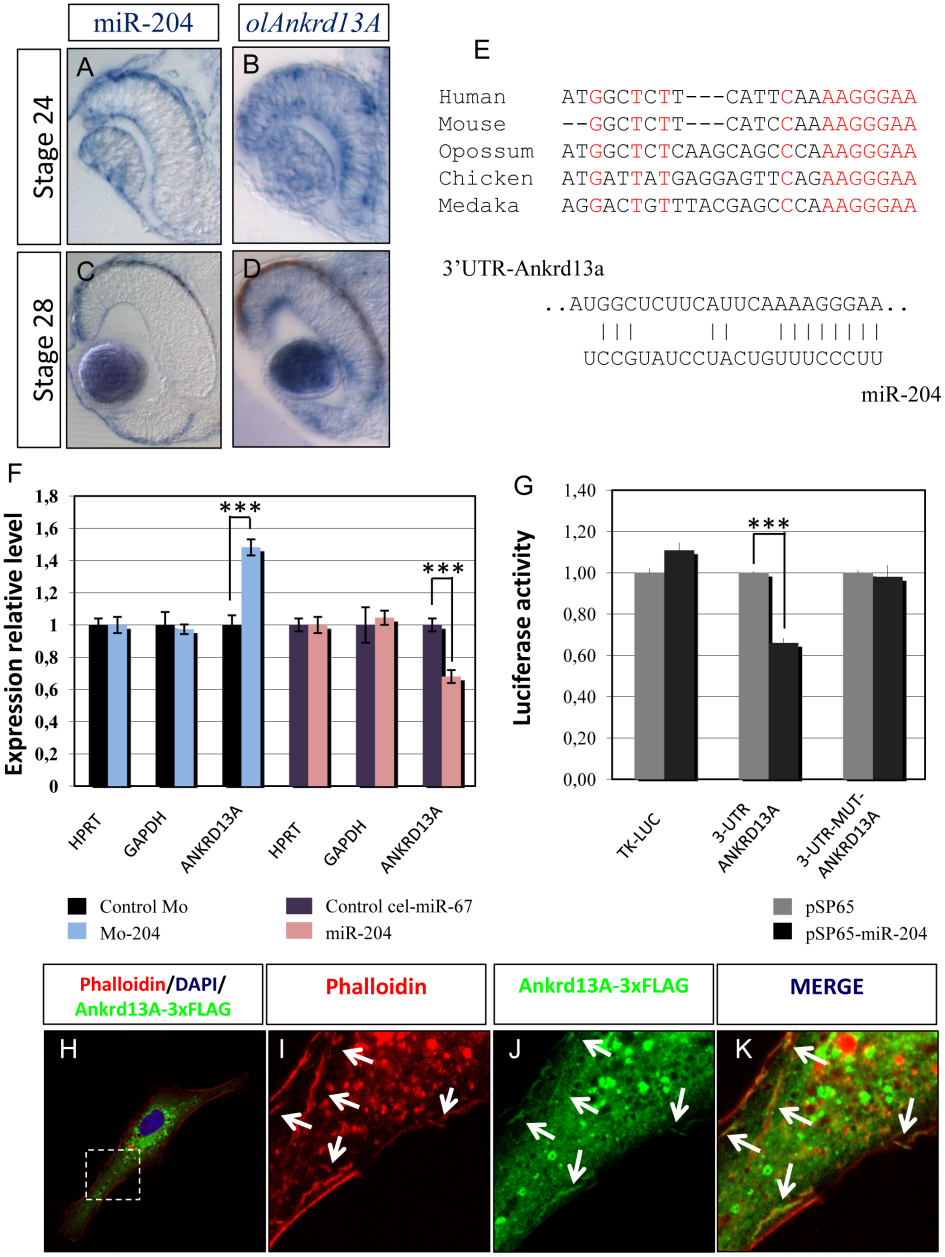
miR-204 directly targets *Ankrd13A*. (A–D) Frontal sections of St24 and St28 WT medaka embryos hybridized in single whole-mount RNA ISH with probes against miR-204 (A, C) and *olAnkrd13A* (B, D). (E) The predicted target site of miR-204 within the 3′-UTR of the *Ankrd13A* gene in different species: conserved nucleotides are marked in red and non-conserved nucleotides are marked in black. (F) Histograms showing the fold change variations (expressed as 2^−ΔΔCt^ values) in the mRNA levels of *Ankrd13A* quantified by qRT-PCR in medaka embryos injected with Mo-miR-204 or miR-204 with respect to controls. The *Hprt* and *Gapdh* genes were used to normalize the results. (G) Relative Luc activities in H36CE cells as fold differences in the Luc/Renilla ratios normalized to the value of Luc reporter constructs. miR-204 addition significantly decreases Luc activity of the construct containing the 3′-UTR of *ANKRD13A* when compared to controls. ***P<0.001 (t tests). Three point mutations in the predicted miR-204 target site in *ANKRD13A* inhibit this effect (no significant variation when compared with the thymidine kinase (TK)- Luc control). (H) Immunofluorescence with a monoclonal antibody against the FLAG tag (red) after short transfection times of FLAG-tagged full-length *ANKRD13A* to minimize overexpression. Boxed area is magnified in I–K. (I–K) Actin was visualized by staining with phalloidin (Red). ANKRD13A (Green) colocalizes with actin filaments (arrows). Notably, the ANKRD13A OE determined the depolymerization of actin filaments.

We then searched for miR-204 potential mRNA targets that may be linked to the control of cell migration. Toward this goal, we compiled a non-redundant list of miR-204 predicted target genes, as assessed from the analysis of the miRanda, TargetScan and PicTar algorithms [31], [32], [33]. A Gene Ontology (GO) analysis of this list, using the DAVID tool (http://david.abcc.ncifcrf.gov/), confirmed that miR-204 predicted targets were significantly enriched for genes involved in cell adhesion and migration (data not shown). We carried out a detailed *in silico* screening of the latter targets, based on their reported expression profiles in the developing mammalian eye and on their predicted function. Notably, among the predicted miR-204 targets, the *Ankyrin repeat domain 13A* [(*ANKRD13A);* NM_033121.1] gene emerged as an interesting candidate target gene to mediate the action of miR-204 in cell migration/adhesion, because: a) it was previously reported to be significantly expressed in the lens [40]; and b) it was predicted to interact with both actin-binding and FA proteins [41]. Accordingly, double immunofluorescence staining showed a significant colocalization of actin microfilaments with the *ANKRD13A* gene product (Figure 3 H–K).

We observed that, in medaka embryos, miR-204 and *olAnkrd13A* display overlapping expression patterns in the lens and in migrating NCCs (Figure 3A–D). Furthermore, the predicted target site of miR-204 within the 3′-UTR of *ANKRD13A* is highly conserved across all vertebrate species analyzed, including medaka (Figure 3E). To validate this prediction, we cloned the 3′-UTR of the human *ANKRD13A* gene, containing the miR-204 target site, downstream of the coding region of the Luciferase (Luc) reporter gene, and tested the ability of miR-204 to affect reporter expression *in vitro*. The presence of the *ANKRD13A* 3′-UTR sequence inhibited Luc activity in response to miR-204 (Figure 3G). Consistently, point mutations in the miR-204 target site abolished the repression, thus indicating that miR-204 directly and specifically targets *ANKRD13A* (Figure 3G). We determined that *ANKRD13A* behaved as a miR-204 target by quantitative Reverse Transcriptase (qRT)-PCR on total RNA derived, respectively, from control, miR-204 KD and miR-204 OE transiently transfected H36CE lens and A549 cells (Fig. S2 and Fig. S4). Furthermore, the miR-204 targeting of *olAnkrd13A* was also confirmed *in vivo*. Injections of miR-204 duplexes in medaka embryos resulted in a decrease of the endogenous *olAnkrd13A* mRNA levels, whereas injections of Mo-miR-204 led to their increase (Figure 3F).

### Ankrd13A Controls Cytoskeleton and FA Organization

**Figure 4:**
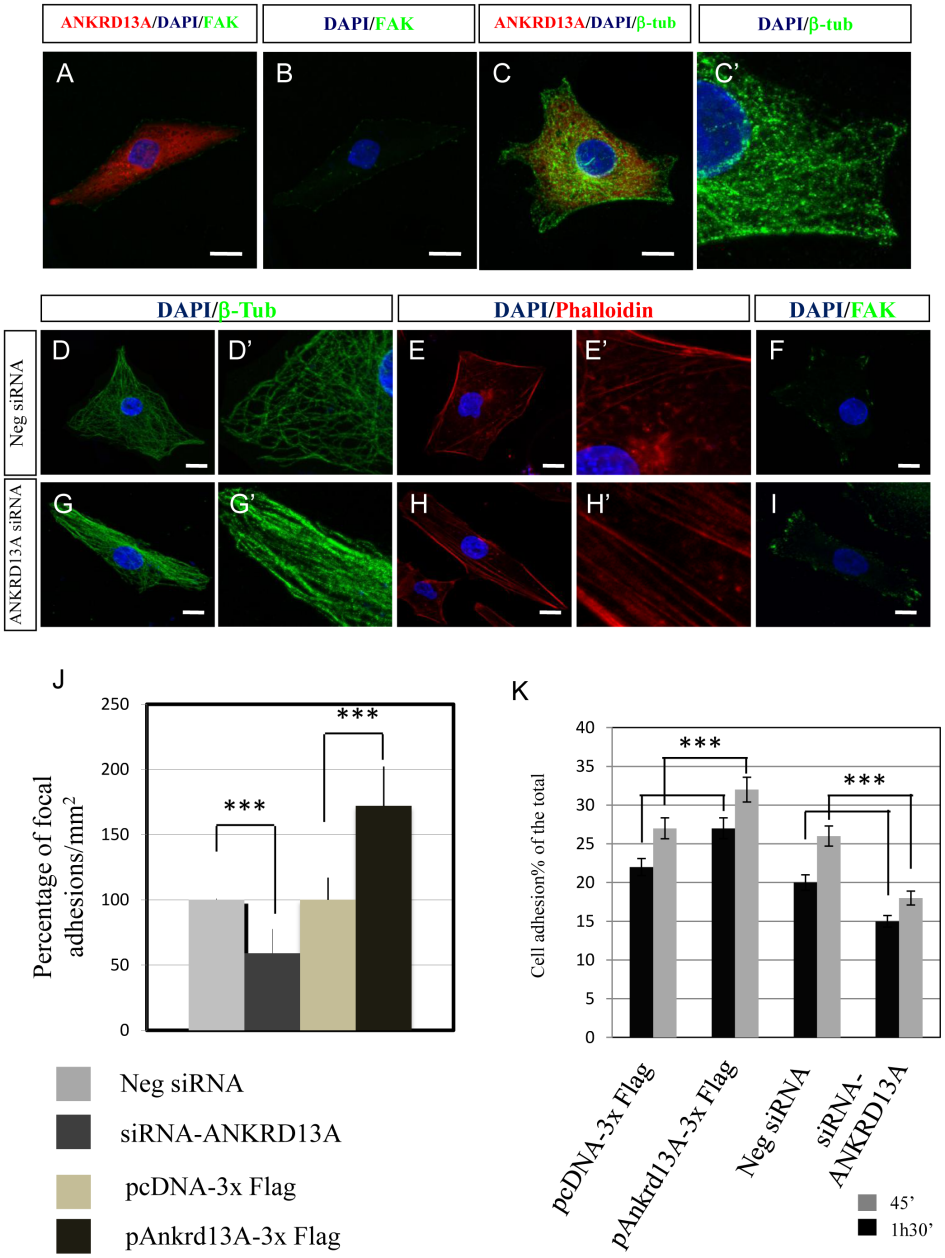
ANKRD13A controls cytoskeleton and FA organization. (A–B) Immunostaining of FAK (green) and nuclei (DAPI-blue) in ANKRD13A-3×Flag (Red) OE H36CE lens cells. (C–C′) Immunostaining of β-tubulin (green) and nuclei (DAPI-Blu) in ANKRD13A-3×Flag (Red) OE H36CE lens cells. Immunostaining of β-tubulin (green), nuclei (DAPI-blu) and actin filaments with phalloidin (Red) in control (D–F), and ANKRD13A KD (G–I) H36CE lens cells. (J) Relative percentage of FA per area in H36CE cells. ANKRD13A OE displays a significant increase in the number of FA per area with respect to control. A marked decrease of both percentage of FA per area and FA polarized distribution was observed in ANKRD13A KD H36CE lens cells. ***P<0.0001 (t tests). (K) Extent of cell adhesion was plotted as a percentage. The number of cell adhesions is significantly increased by ANKRD13A OE and significantly decreased by ANKRD13A KD in H36CE lens cells. ***P<0.0001 (t tests).

Ankyrin repeat domain-containing (Ankrd) proteins are a large family of molecules, involved in protein-protein interactions and are implicated in a variety of different cellular functions [42]. Interestingly, a member of this family, named *Ankrd28* [*NM_001195098.1*], has been recently found to contribute to the modulation of cell migration [43], [44]. Therefore, we asked whether miR-204-mediated control of *Ankrd13A* contributes to the control of cell migration. To test this hypothesis, we sought to assess the relative contribution of the *ANKRD13A* gene product to the miR-204-mediated control of cell adhesion and migration by studying the effects of *ANKRD13A* KD or OE on H36CE cell migration. Strikingly short-interfering RNA (siRNA)-mediated *ANKRD13A* KD (Figure S5A), determined a significant increase in the speed of cell migration compared to control cells (Figure S5C, D, E, F). Conversely, *ANKRD13A* OE (Figure S5B) caused the opposite behavior resulting in a significant decrease of cell migration (Figure S5G, H, I, J). Similar to what previously described in response to miR-204 KD, we found that *ANKRD13A* OE induced significant disassembly of fasciculated actin, radial distribution of microtubules and increase of both FA distribution and cellular adhesion (Figure 4A–C′, J–K). Opposite effects were observed in *ANKRD13A* KD cells, which exhibited fasciculated microtubules and actin fibers, and polarized distribution of FA (Figure 4D–I, J–K). Altogether, our data demonstrate that *ANKRD13A* plays a key role in cytoskeleton and FA organization.

### *miR.204* Controls Cell Motility by Targeting Ankrd13A

**Figure 5:**
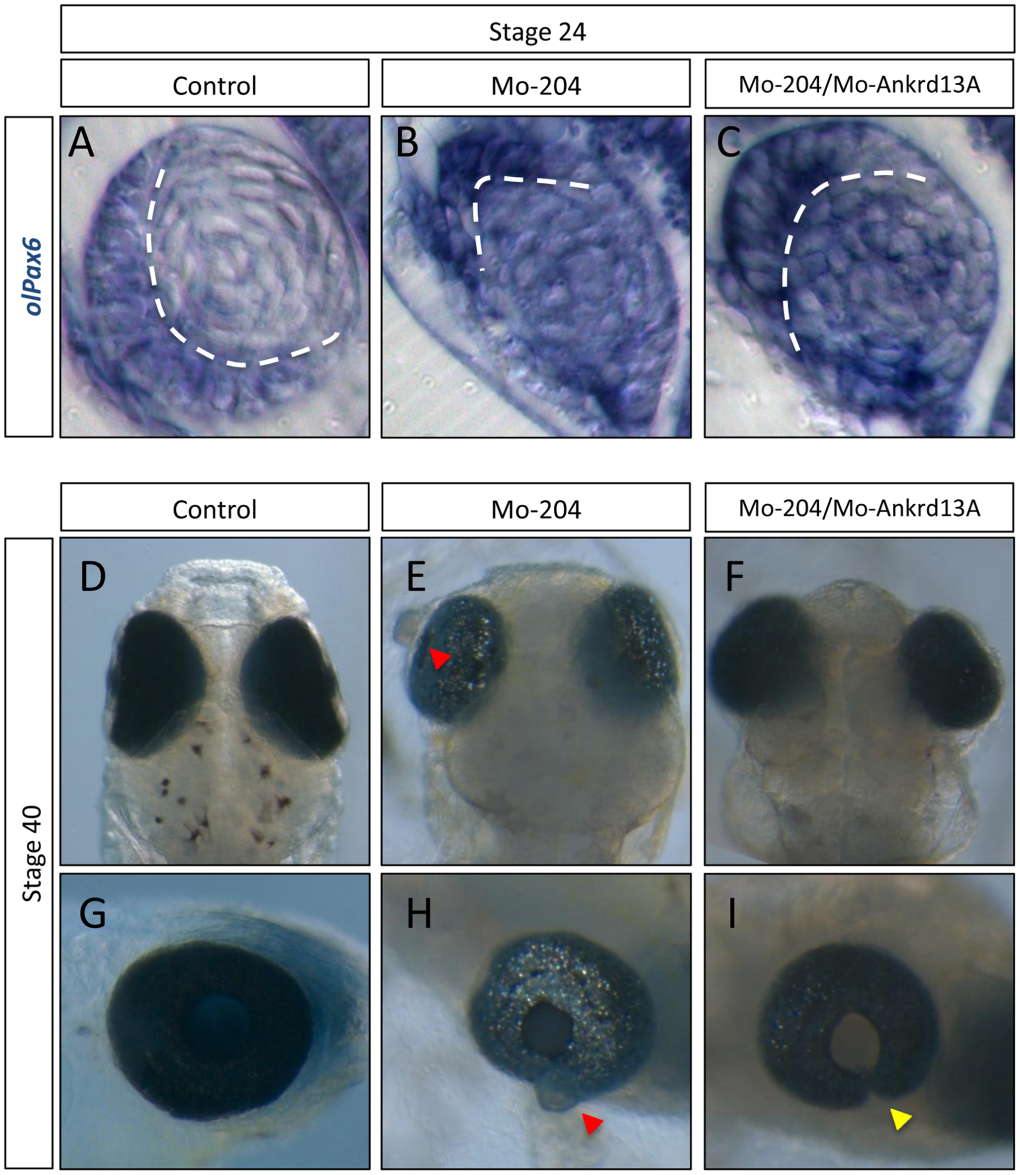
Ankrd13A depletion specifically rescues the miR-204 morphant lens phenotype. Frontal sections of St24 control (A), MO-miR-204 (B), and MO-miR-204/Mo-Ankrd13A (C)-injected medaka embryos processed for whole-mount RNA ISH with an *olPax6* probe (A–C). Notably, interference with *Ankrd13A* expression fully recovers primary fiber lens cell mislocalization, but not lens epithelial cell localization that are dorsally located. Dashed lines mark the boundaries between the lens epithelial monolayer and the primary fiber cells. Bright-field stereomicroscopy images of St40 control (D,G), Mo-miR-204 (E,H), Mo204/Mo-Ankrd13A (F,I) injected medaka embryos as dorsal (D, E, F), and lateral (G, H, I) views. In Mo-miR-204/Mo-Ankrd13A-injected embryos, the lens phenotype is rescued. Notably, the lens does not protrude out of the optic cup (F, I, yellow arrows) when compared to miR-204 morphant embryos (E, H, red arrows).

To determine whether the misregulation of *Ankrd13A* was linked to the Mo-miR-204 phenotype observed *in vivo,* we co-injected Mo-miR-204 with a morpholino (Mo) against *olAnkrd13A* (Mo-Ankrd13A) to counteract the *olAnkrd13A* upregulation. We found that Mo-Ankrd13A injection was sufficient to rescue the normal distribution of lens fiber cells that formed organized concentrical layers in a substantial proportion of miR-204 morphant embryos (87±5% of 2,300 injected embryos; Figure 5A–C). Remarkably, Mo-Ankrd13A counterbalanced the formation of lens herniation induced by the reduction of miR-204 activity (Figure 5D–I). These data confirmed, *in vivo*, that the *Ankrd13A* gene is targeted by miR-204 and is involved in the modulation of lens fiber cell migration (Figure 5A–C). The reduced levels of miR-204 also interfered with the establishment of a proper dorso/ventral and proximo/distal polarity of lens patterning (Figure 5A–C). However, this miR-204 activity is very likely to be Ankrd13A-independent, because reduction of *Ankrd13A* expression levels did not rescue the anomalies of lens polarity (Figure 5A–C). This finding indicates the existence of additional and as yet unidentified miR-204-dependent mechanisms implicated in lens patterning. Altogether, these data indicate that miR-204 controls lens fiber cell motility by modulating the expression levels of the *Ankrd13A* gene product.

## Discussion

We previously showed [13] that miR-204 inactivation in medaka led to a) aberrant lens herniation, b) malposition of the monolayer of epithelial cells in the dorsal region of the lens vesicle, and c) misplacement of primary fiber cells in the center of the lens vesicle and lack of their elongation to form organized concentrical layers (Figure S1). In this study, we carried out a deeper analysis of the above phenotype. In particular, we found that in miR-204 morphant embryos, NCCs in the dorsal migratory stream fail to correctly migrate anteriorly. Time-lapse analysis of cell behaviors demonstrated that these cells have significantly reduced velocity and cellular protrusion. Conversely, miR-204 overexpression led to a significant increase of cell motility and length of cellular projections (Figure 1). We then concluded that miR-204 contributes to the organization of lens tissue by regulating cell motility. In analyzing this phenotype, we also uncovered a new role of miR-204 in the control of mesenchymal NCC migration and we provided solid support to the contribution of microRNAs to mesenchymal cell migration processes *in vivo*.

We have further characterized the mechanism of miR-204-dependent control of cell motility by identifying the *Ankrd13A* transcript as a direct functional target of miR-204 (Figure 3 and 4). In particular, we found that *Ankrd13A* modulates the lens cell migration program by interfering with both cytoskeleton and focal adhesion formation (Figure 4). *Ankrd13A* is a member of the ankyrin repeat domain-containing protein family and its role is largely unknown. However, Ankrd13A has been recently implicated in endocytosis on the basis of its ability to inhibit the ubiquitylation-dependent internalization of the ligand-activated EGF receptor (EGFR) [45]. Given the interaction of EGFR with Focal Adhesion Kinase (FAK) in promoting cell migration [46], it will be interesting to dissect the mechanisms that link miR-204 and Ankrd13A to the control of EGF-induced cell migration, in both physiological and pathological conditions. Interestingly, ANKRD28, a member of ankyrin repeat domain-containing protein family was linked as a scaffold protein to assemble components of focal adhesion proteins, such as 130Cas-Crk-DOCK180 complex in the trailing edges of the migratory cells [43], [44]. These data also strongly argue for the physiological relevance of this protein family in participating to cell migration.

It is important to point out that miR-204 has a closely related paralog, i.e., miR-211, in mammals. MiR-204 and miR-211 share the same seed-region sequence and only differ by one or two nucleotides, depending on the species. MiR-204 and miR-211 have been classified as a single subfamily of miRNAs that share the same set of predicted targets (TargetScan) [31].The evolution of the miR-204/211 subfamily is quite interesting. MiR-204 is present in two identical copies in the genomes of early vertebrates and fish, including medaka fish [13]. MiR-211 first appears in mammals by the evolution of one of the two copies of miR-204. Therefore, the use of the medaka fish as model organism to study the function of miR-204 has the notable advantage of providing information on the function of the subfamily of miR-204/211 as a whole. On the other hand, it does not allow to identify possible differences in the specific functional roles of either of the two miRNAs, including the control of cell migration. As a consequence, it will be necessary in the near future to carry out additional and specific assays separately for the two miRNAs in mammalian models to dissect their specific functional roles.

Interestingly, these two miRNAs have been studied in a wide variety of cancer, RPE and lens cell lines, and their activities *in vitro* has been documented and associated with cell migration, differentiation and invasiveness [15], [16], [18], [19], [20], [47], [48], [49], [50], [51], [52]. However, different and contradictory phenotypes were associated with the inactivation and/or overexpression of these miRNAs in different cell lines. Increased expression of miR-204 and miR-211 was found to reduce migration and invasion in some human melanoma and lens cell lines [16], [48], [50], [51] while it was reported to enhance both motility and invasiveness in other human melanoma and breast cancer cells [20], [48]. Based on the above considerations, it is possible that miR-204/211 activity in modulating cell motility is dependent on the properties of the cellular context, such as the presence or absence of their target mRNAs and the concomitant action of other miRNAs. This functional diversity has already been demonstrated in the case of another miRNA, i.e., miR-10b, which, although ubiquitously expressed, was shown to exhibit different functions, depending on the repertoire and stoichiometry of its direct mRNA targets in different cellular contexts [53]. Notably, our analysis of the different effects of miR-204 on TGFB1-treated (mesenchymal-like) and untreated (epithelial-like) lung tumor-derived A549 cells supports a highly specific role of the miR-204 in controlling cell motility in a context-dependent fashion, by affecting cell types already primed for migration.

**Figure 6:**
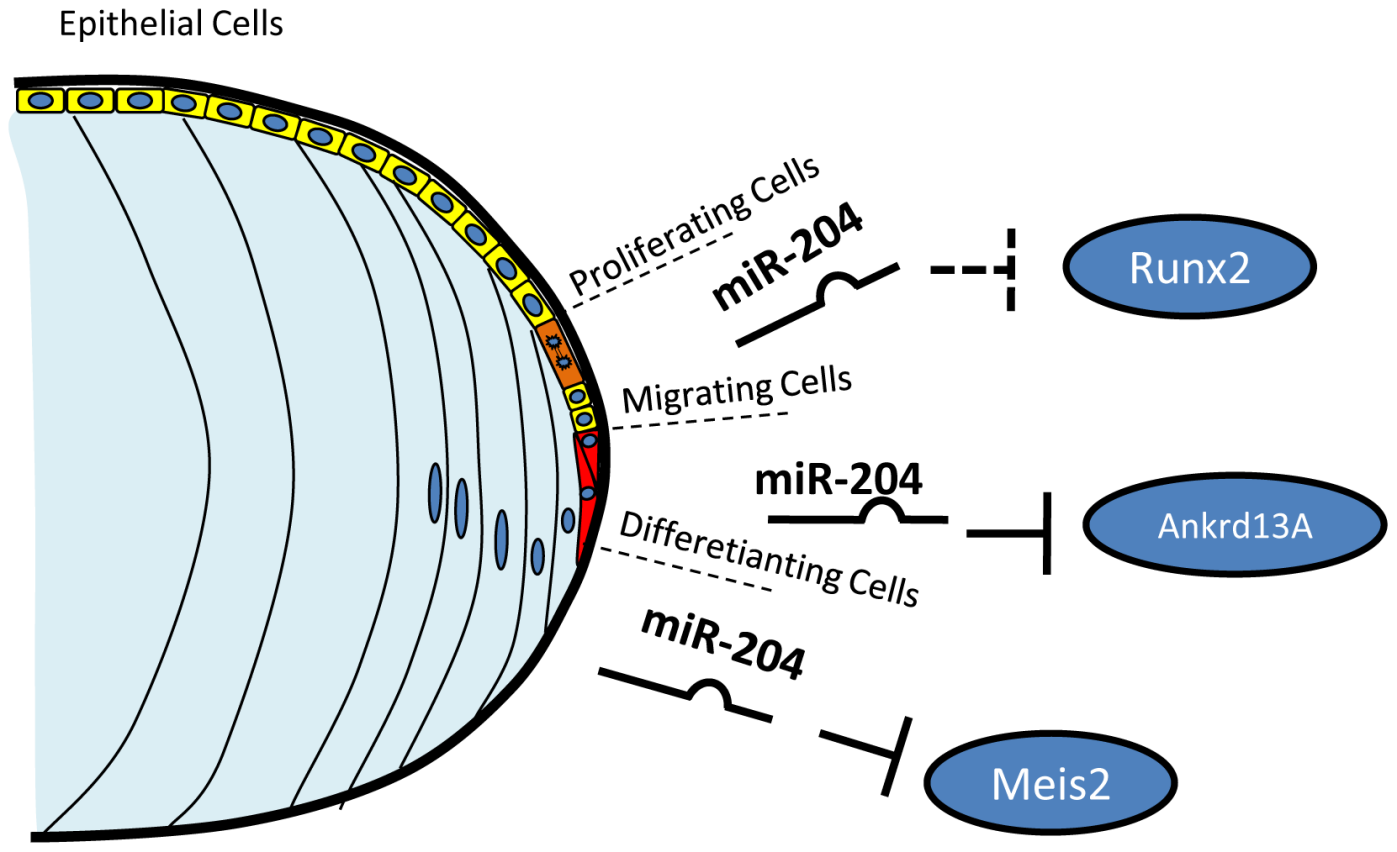
Schematic model of the proposed function of miR-204 in lens development. MiR-204 acts in a context-dependent manner in proliferating epithelial, migrating, and differentiating lens cells. In epithelial cells, miR-204 controls the expression of the *Runx2* gene, contributing to the regulation of the proliferative pathway [50]. In migrating cells, miR-204 activity regulates fiber lens cell migration by targeting *Ankrd13A* gene and modulating FA and cytoskeleton organization (this paper). In differentiating lens cells, miR-204 modulates the *Meis2/Pax6* gene pathways and the related cell differentiation molecular networks [13].

The data presented in this study, along with previously reported findings, strongly indicate that miR-204 plays a role in multiple aspects of lens development. Lens differentiation is a quite complex process. In the first stages of lens development, two main types of cells differentiate from the primordial lens vesicle. Cells localized in the posterior half of the vesicle are committed to differentiate into primary lens fibers, whereas cells in the anterior part of the vesicle differentiate into epithelial cells. Later on, the lens grows by cell division and differentiation in the equatorial (transitional) zone and new fiber cells are continuously added to the fiber mass. The progeny of epithelial cells, after a number of cell divisions, migrate, elongate and differentiate into lens fiber cells (Figure 6) [54]. Timely differentiation and correct migration of lens fibers are crucial for continuous addition of fiber mass and formation of a correctly organized lens. Our present data, along with previously reported evidence, indicate that miR-204 participates, in a context-dependent manner, to the main phases of lens development by modulating the expression of several key target genes: 1) *Runx2* [NM_009820.4], in the regulation of cell proliferation in the lens epithelial compartment [50]; 2) *Ankrd13A*, in the control of cell migration in the lens transitional zone; 3) *Meis2/Pax6,* in the modulation of lens cell differentiation and patterning [13].

### Conclusions

Our study uncovered a novel role of miR-204 in cell migration in a vertebrate model organism, as determined through the assessment of its involvement in both lens and NCC cell motility. This finding, together with the previous observations that miR-204 is also involved in proliferation [50] and differentiation of lens cells [13], constitutes an additional step ahead towards the full identification of the miR-204-controlled molecular network. This, in turn, might help to define the molecular basis of human eye defect, such as, for instance, secondary cataract, which has already been associated with alterations of the miR-204 expression [50]. In summary, miR-204, due to its involvement in all main aspects of lens development, may be considered a “master regulator” of the molecular networks that regulate lens formation in vertebrates.

## Supporting Information

Figure S1 **miR-204 knockdown determines mis-localization of mesenchymal cells that express miR-204.** Comparison by RNA in situ hybridization (ISH) of the expression profiles of miR-204 host genes in medaka, i.e., *olTrpm1* and *olTrpm3*, which are co-expressed with miR-204 (Karali et al. 2007). Frontal sections of St24 control (A–B) and Mo-miR-204 (C–D) injected medaka embryos hybridized with *olTrpm1* (A, C) and *olTrpm3* (B, D) probes. *olTrpm1* is normally expressed in both mesenchymal lens fiber cells (black arrows) and neural crest migrating cells (red arrows) whereas *olTrpm3* is only expressed in mesenchymal lens fiber cells (B). In miR-204 morphant embryos, both the above cell types are mis-localized (C, D). (E) Dorsal view of a wild-type medaka embryo hybridized with an *olTRPM1* RNA probe. (F) Dorsal view of a *olTRPM1*:EGFP transgenic medaka embryo. Note how GFP expression in the transgenic line recapitulates the endogenous *olTRPM1* gene expression.
(TIF)

Figure S2 **miR-204 modulates the motility of H36CE cells.** (A–J) Images of H36CE lens cells at 0 and 48 h after wounding. A wound scratch was introduced in confluent monolayers of: wild-type (A), control inhibitor cel-miR67-transfected (C), inhibitor hsa-miR-204-transfected (E), control mimic cel-miR67-transfected (G), mimic hsa-miR-204-transfected (I) H36CE cells. (B, D, F, H, J) After 48 hrs, there are still extensive gaps in the wound edge of miR-204 depleted H36CE cells (F), while miR-204 overexpressing H36CE lens cells have migrated more into the gap to close the wound (J) in comparison to controls (B, D, H). (K) Tracking the position of the advancing wound edge revealed a significant decrease in the speed of wound closure in the inhibitor hsa-miR-204 transfected H36CE cells. A marked increase in the speed of wound closure was present in the mimic hsa-miR-204 transfected H36CE cells. ***P<0.0001 (t tests). (J) Histograms showing the fold change variations (expressed as 2-ΔΔCt values) in the levels of the *ANKRD13A* mRNA quantified by qRT-PCR in H36CE cells transfected with miR-204 mimic or miR-204 inhibitor reagents with respect to control- (cel-miR-67) transfected cells. Note that the expression of *ANKRD13A* is decreased in cells transfected with the miR-204-mimic while it is increased in cells treated with the miR-204 inhibitor. (L) Histograms showing the fold change variations (expressed as 2-ΔΔCt values) of the miR-204 level quantified by TaqMan qRT-PCR in H36CE cells transfected with miR-204 mimic. Note that the expression of miR-204 is increased in cells treated with miR-204 mimic.
(PPTX)

Figure S3 **Cell cycle is not altered in H36CE human lens cells following miR-204 expression perturbation.** (A, B) Histograms showing the fold change variations (expressed as 2-ΔΔCt values) in the mRNA levels of HPRT, GAPDH, CHORDC1, PAX6, MEIS2, α-ACRYSTALLIN, PROX1 and SOX2 quantified by qRT-PCR in H36CE cells transfected with miR-204 mimic (A) or miR-204 inhibitors (B) with respect to control (cel-miR-67) transfected cells. Note that expression of MEIS2, PAX6, PROX1 and α-ACRYSTALLIN is increased in cell transfected with miR-204-mimic while it is decreased in cells treated with miR-204 inhibitor. The HPRT, CHORDC1 and GAPDH housekeeping genes were used to normalize the expression of the analyzed genes. (C) Histograms showing the cell cycle profile of H36CE cells transiently transfected with miR-204 mimic or miR-204 inhibitor reagents with respect to control (cel-miR-67) transfected cells. (D) Average percentages are shown for G_0_/G_1_, S and G2/M DNA content. The cell-cycle profiles are not affected after miR-204 expression alteration.
(PPTX)

Figure S4 **miR-204 modulates the motility of A549 cells.** (A–D) Images of A459 epithelial cells at 0 and 48 h after wounding. A wound scratch was introduced in confluent monolayers of (A) control mimic cel-miR67-transfected (C), mimic hsa-miR-204-transfected A459 epithelial cells. (B, D) After 48 hrs, there were no differences in the wound edge of miR-204-OE A459 epithelial cells (D) in comparison to control cells (B). (E–H) Images of A459 mesenchymal cells, i.e., treated with TGFB1, at 0 and 30 h after wounding. After 30 hrs, miR-204 -OE A459 mesenchymal cells have migrated more into the gap to close the wound (H) in comparison to controls (I). (K) Histograms showing the fold change variations (expressed as 2-ΔΔCt values) of miR-204 levels quantified by TaqMan qRT-PCR in both A549 epithelial and mesenchymal cells transfected with a miR-204 mimic. Note that the expression of miR-204 is increased in cells treated with miR-204 mimic. (J) Histograms showing the fold change variations (expressed as 2-ΔΔCt values) in the level of the ANKRD13A mRNA quantified by qRT-PCR in A549 cells transfected with miR-204 mimic with respect to control- (cel-miR-67) transfected cells. Note that the expression of *ANKRD13A* is decreased in cells transfected with the miR-204-mimic. (L) Tracking the position of the advancing wound edge revealed a significant increase in the speed of wound closure in the hsa-miR-204 transfected A459 mesenchymal cells whereas there was no difference in the speed of A549 epithelial cells. ***P<0.0001 (t tests). (M) Immunoblot analysis of VIMENTIN and E-CADHERIN in epithelial and mesenchymal TGF beta-treated A459 cells.
(PPTX)

Figure S5 **The** ***ANKRD13A*** **gene has an impact on wound healing capacity** ***in vitro*** **.** (A) H36CE cells were lysed 48 h after transfections of an *ANKRD13A* siRNA or of a vehicle alone, and mRNA levels were quantified by qRT-PCR. (B) Cells were lysed 48 h after transfections of ANKRD13-3×Flag or pcDNA3xFlag vectors, and amounts of protein were quantified by Western blots. (C–J) Images of cells at 0 and 48 h after wounding. A wound scratch was introduced in confluent monolayers of control negative siRNA-transfected (C), *ANKRD13A* siRNA-transfected (E), control vector pcDNA3xFlag-transfected (G), and ANKRD13A-3xFlag-transfected (I) H36CE lens cells. (D, F, H, J) After 48 hrs, *ANKRD13A*-depleted H36CE cells (F) have migrated into the gap to close the wound, while there are still extensive gaps in the wound edge of ANKRD13A-3×Flag overexpressing H36CE lens cells (J) in comparison to controls (D and H). (K) Tracking the position of the advancing wound edge revealed a significant increase in the speed of wound closure in the *ANKRD13A* siRNA-transfected H36CE cells whereas there was a marked decrease in the speed of wound closure in the ANKRD13A-3×Flag transfected H36CE cells. Data are means ± SEM of values; ***P<0.0001 (t tests).
(TIF)

Table S1 **Primers and Morpholinos Sequences.**
(XLS)

Movie S1 **Timelapse movie of olTrpm1:GFP-marked neural crest migrating cell in control medaka embryos.** The movie shows the migration of individual olTrpm1:GFP-marked neural crest migrating cell in control medaka embryos. The cell covers a distance of 205 µm in 8 hours, see also Figure 1 for details.
(MOV)

Movie S2 **Timelapse movie of olTrpm1:GFP-marked neural crest migrating cell in miR-204morphant medaka embryos.** The movie shows the migration of individual olTrpm1:GFP-marked neural crest migrating cell in miR-204 morphant medaka embryos. The cell covers a distance of 150 µm in 8 hours, see also Figure 1 for details.
(MOV)

Movie S3 **Timelapse movie of olTrpm1:GFP-marked neural crest migrating cell in miR-204 overexpressing medaka embryos.** The movie shows the migration of individual olTrpm1:GFP-marked neural crest migrating cell in miR-204 overexpressing medaka embryos. The cell covers a distance of 205 µm in 5 hours, see also Figure 1 for details.
(MOV)
