# Supplementary figures and images for: miR-204 Targeting of Ankrd13A Controls Both Mesenchymal Neural Crest and Lens Cell Migration
Source: PLoS One. 2013 Apr 19;8(4):e61099. doi: 10.1371/journal.pone.0061099 (PMC3631221; doi:10.1371/journal.pone.0061099)

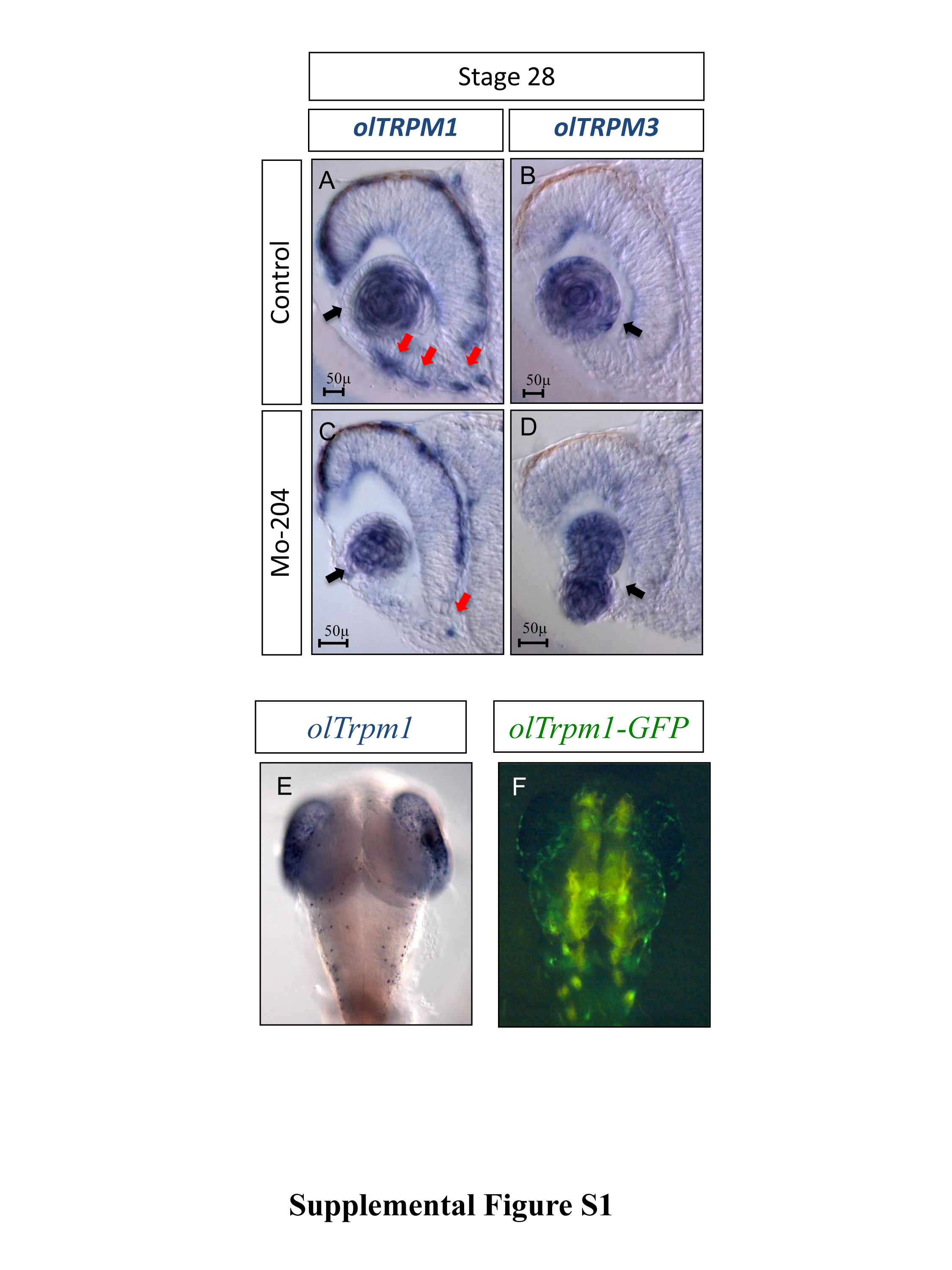

Supplement: Figure S1 — miR-204 knockdown determines mis-localization of mesenchymal cells that express miR-204. Comparison by RNA in situ hybridization (ISH) of the expression profiles of miR-204 host genes in medaka, i.e., olTrpm1 and olTrpm3, which are co-expressed with miR-204 (Karali et al. 2007). Frontal sections of St24 control (A–B) and Mo-miR-204 (C–D) injected medaka embryos hybridized with olTrpm1 (A, C) and olTrpm3 (B, D) probes. olTrpm1 is normally expressed in both mesenchymal lens fiber cells (black arrows) and neural crest migrating cells (red arrows) whereas olTrpm3 is only expressed in mesenchymal lens fiber cells (B). In miR-204 morphant embryos, both the above cell types are mis-localized (C, D). (E) Dorsal view of a wild-type medaka embryo hybridized with an olTRPM1 RNA probe. (F) Dorsal view of a olTRPM1:EGFP transgenic medaka embryo. Note how GFP expression in the transgenic line recapitulates the endogenous olTRPM1 gene expression. (TIF) [file pone.0061099.s001.tif]

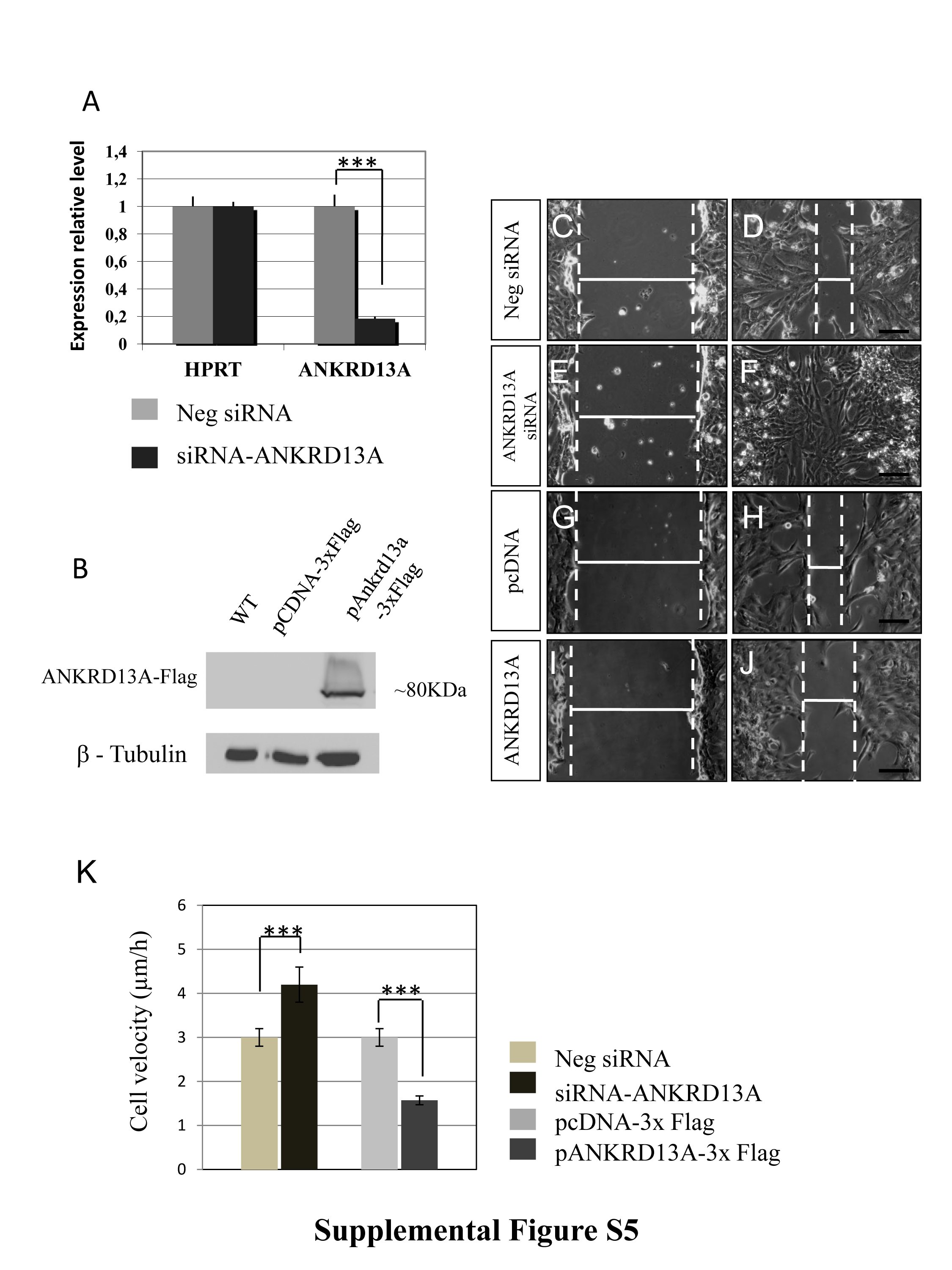

Supplement: Figure S5 — The ANKRD13A gene has an impact on wound healing capacity in vitro . (A) H36CE cells were lysed 48 h after transfections of an ANKRD13A siRNA or of a vehicle alone, and mRNA levels were quantified by qRT-PCR. (B) Cells were lysed 48 h after transfections of ANKRD13-3×Flag or pcDNA3xFlag vectors, and amounts of protein were quantified by Western blots. (C–J) Images of cells at 0 and 48 h after wounding. A wound scratch was introduced in confluent monolayers of control negative siRNA-transfected (C), ANKRD13A siRNA-transfected (E), control vector pcDNA3xFlag-transfected (G), and ANKRD13A-3xFlag-transfected (I) H36CE lens cells. (D, F, H, J) After 48 hrs, ANKRD13A-depleted H36CE cells (F) have migrated into the gap to close the wound, while there are still extensive gaps in the wound edge of ANKRD13A-3×Flag overexpressing H36CE lens cells (J) in comparison to controls (D and H). (K) Tracking the position of the advancing wound edge revealed a significant increase in the speed of wound closure in the ANKRD13A siRNA-transfected H36CE cells whereas there was a marked decrease in the speed of wound closure in the ANKRD13A-3×Flag transfected H36CE cells. Data are means ± SEM of values; ***P<0.0001 (t tests). (TIF) [file pone.0061099.s005.tif]
